# Emodin alleviates intestinal ischemia–reperfusion injury through antioxidant stress, anti-inflammatory responses and anti-apoptosis effects via Akt-mediated HO-1 upregulation

**DOI:** 10.1186/s12950-024-00392-z

**Published:** 2024-07-09

**Authors:** Yinyin Liu, Tuo Ji, Haixing Jiang, Meng Chen, Wanli Liu, Zongze Zhang, Xianghu He

**Affiliations:** 1 Department of Anesthesiology, Zhongnan Hospital of Wuhan University, East Lake Road, Wuhan, Hubei 430071 China; 2 Department of Anesthesiology, School and Hospital of Stomatology, Wuhan University, Wuhan, Hubei 430079 China; 3 Department of Anesthesiology, Hubei Maternal and Child Health Hospital, Wuhan, Hubei 430070 China; 4 Department of Anesthesiology, Jiayu Hospital, Zhongnan Hospital of Wuhan University, Xianning, Hubei 437200 China

**Keywords:** Intestinal ischemia–reperfusion injury, Antioxidant stress, Anti-inflammation, Anti-apoptosis, Akt/HO-1 signaling pathway

## Abstract

**Background:**

Intestinal ischemia–reperfusion (I/R) injury is a severe vascular emergency. Previous research indicated the protective effects of Emodin on I/R injury. Our study aims to explore the effect of Emodin on intestinal I/R (II/R) injury and elucidate the underlying mechanisms.

**Methods:**

C57BL/6 mice and Caco-2 cells were used for in vivo and in vitro studies. We established an animal model of II/R injury by temporarily occluding superior mesenteric artery. We constructed an oxygen–glucose deprivation/reoxygenation (OGD/R) cell model using a hypoxia-reoxygenation incubator. Different doses of Emodin were explored to determine the optimal therapeutic dose. Additionally, inhibitors targeting the protein kinase B (Akt) or Heme oxygenase-1 (HO-1) were administered to investigate their potential protective mechanisms.

**Results:**

Our results demonstrated that in animal experiments, Emodin mitigated barrier disruption, minimized inflammation, reduced oxidative stress, and inhibited apoptosis. When Akt or HO-1 was inhibited, the protective effect of Emodin was eliminated. Inhibiting Akt also reduced the level of HO-1. In cell experiments, Emodin reduced inflammation and apoptosis in the OGD/R cell model. Additionally, when Akt or HO-1 was inhibited, the protective effect of Emodin was weakened.

**Conclusions:**

Our findings suggest that Emodin may protect the intestine against II/R injury through the Akt/HO-1 signaling pathway.

## Introduction

Ischemia–reperfusion (I/R) injury refers to the pathological condition in which tissue or organ damage occurs as a result of the temporary interruption and subsequent restoration of blood flow. The intestine is particularly susceptible to I/R injury, and its detrimental effects can extend beyond the intestine, affecting distant tissues and organs. Ischemic insult to the intestine increases microvascular permeability and disrupts the mucosal barrier. Reperfusion leads to an influx of oxidative products and inflammatory cell infiltration [1]. If the disrupted barrier exceeds its regenerative capacity, it can lead to sepsis and organ failure, underscoring its clinical significance [2].

Oxidative stress plays a crucial role as a mediator in the complex pathogenesis of intestinal I/R (II/R) injury [3]. The development of II/R injury is mediated by critical factors such as oxygen free radicals, leukocyte infiltration, inflammatory mediators, and disruption of energy metabolism [4, 5]. Meanwhile, apoptosis inevitably plays an important role in almost all pathological processes of I/R injury [6]. Research efforts have also focused on developing strategies to reduce inflammation, and oxidative stress [7]. The pathogenesis of II/R injury involves multiple signaling pathways, including MAPK signaling, NF-κB signaling, and Nrf2 signaling [8, 9]. Since preventing intestinal ischemia is challenging, it’s necessary to study the mechanism to alleviate intestinal injury.

Traditional Chinese medicine has unique advantages in improving the intestinal prognosis of II/R injury due to its multifunctional characteristics. Emodin (1,3,8-trihydroxy-6-methylanthraquinone), a bioactive compound functioning as a natural anthraquinone aglycone, can be extracted from herbaceous species in the Fabaceae, Polygonaceae, and Rhamnaceae families. It is known for its anti-inflammatory and antioxidant properties [10]. Meanwhile, Emodin may act by regulating oxidative stress, cell apoptosis, and endoplasmic reticulum stress to prevent I/R injury [11, 12]. Previous studies have demonstrated the neuroprotective effects of Emodin against I/R injury in the cerebral and retinal tissues [13, 14]. Recent evidence suggests that Emodin may also have the potential to protect against intestinal damage caused by chemicals [15]. But no study has yet shown the relationship between Emodin and II/R injury. The underlying mechanism of Emodin is even less studied.

Protein kinase B (Akt) is a serine/threonine kinase that serves as the central mediator of the PI3K pathway and plays a crucial role in various cellular processes, such as glucose metabolism, apoptosis, cell proliferation, transcription, and cell migration [16]. As a classical anti-apoptotic pathway, the PI3K/Akt signaling pathway is widely studied in I/R injury research [17, 18]. Currently, Akt has also been found to attenuate II/R injury in mice [19, 20].

Furthermore, Heme oxygenase (HO) functions as a rate-limiting enzyme that catalyzes the conversion of heme to biliverdin Ixα, carbon monoxide (CO), and iron. As a target gene that is regulated by nuclear factor E2 p45-related factor 2 (Nrf2), heme oxygenase 1 (HO-1) plays a significant role in responding to antioxidant stress response and ensures cellular protection. It has been reported that the activation of the HO-1/CO signaling pathway is involved in autophagy and the transcription of inflammatory genes [21]. To sum up, Nrf2 is a transcription factor that regulates the anti-oxidative stress response and plays a crucial role in preventing II/R injury by controlling the expression of genes encoding HO-1 and other anti-oxidative enzymes [5, 22]. However, it still needs to be verified whether Emodin regulates Akt/HO-1 signaling pathway in II/R injury.

The present study aims to explore the effect of Emodin on II/R injury and to elucidate the precise mechanisms against II/R injury in vivo and in vitro. This research will shed light on potential targets for clinical therapeutic strategies.

## Materials and methods

### Experimental animals

In this study, 72 male C57BL/6 mice, aged 7–10 weeks and weighing 22-25 g, were used. The mice were obtained from the Animal Feeding Center at the Hubei Province Disease Control Center. And they were sacrificed by isoflurane overdose and immediately decapitated to ensure euthanasia before sample collection. The experimental procedures followed the regulations outlined in the Guide for Laboratory Animal Care and Use by the United States National Institutes of Health and were approved by the Institutional Animal Care and Use Committee of Zhongnan Hospital of Wuhan University, China (No. ZN2023126).

### Establishment of II/R injury animal model

The mice were maintained under optimal conditions for 7 days, including humidity (65–69%), temperature (22–24℃), and a light/dark cycle of 12 h/12 h. Before establishing experiments, animals were kept fasting for 12 h and had free access to water. We put the mice in a special chamber and anesthetized them by using 2–3% isoflurane. The II/R protocol followed previous experiments, involving the temporary occlusion of the SMA using a vascular clamp for 45 min to induce intestinal ischemia. Afterward, the clamp was released, allowing for a 120-min reperfusion period. Small intestinal tissue samples were collected under anesthesia, and the successful model establishment was confirmed by observing the color change of the small intestinal segment.

### Experimental cells

The human colon adenocarcinoma cell line Caco-2 was obtained from the National Collection of Authenticated Cell Cultures in Shanghai, China. Cells were grown in RPMI-1640 Medium GlutaMax-1 supplement (G-4531, Servicebio, Wuhan), supplemented with 10% (v/v) fetal bovine serum and 50 mg/L of penicillin and streptomycin, in a humidified atmosphere of 95% air and 5% CO_2_ at 37 °C. The culture medium was changed every other day, and the cells were typically split at a 1:4 ratio once when they reached confluence.

### Establishment of OGD/R cell model

To establish OGD/R cell model, we used a sugar-free medium (Servicebio, Wuhan) to culture human Caco-2 cells in a three-gas cell incubator with 94% N_2_, 4% CO_2_ and 1% O_2_ for 12 h and subsequently transferred them into normal medium with 95% air and 5% CO_2_ for 4 h.

### Preparations of the Emodin solution and pre-treatment with Emodin

Emodin-treated experimental mice were randomly divided into the following groups (6 mice per group): low-dose Emodin group (20 mg/kg), middle-dose Emodin group (40 mg/kg), and high-dose Emodin group (80 mg/kg) [23] to further investigate the role of Emodin in II/R injury. We consulted the literature and found that repeated pre-treatment with Emodin significantly attenuated damage [24, 25]. We dissolved Emodin (MedChemExpress, USA, HY-B0627) in distilled water with 0.5% sodium methylcellulose according to the manufacturer's instructions for in vivo experiments. The mice in the Emodin group were then given Emodin at various doses via intragastric administration for a duration of 5 days before they underwent the II/R injury. This dosing regimen was chosen based on the bioavailability, metabolism, and intervention cycle reported by Wang et al. [26].

In addition, for in vitro experiments, Emodin was dissolved in dimethyl sulfoxide (DMSO, Sigma, Louis, MO, USA). Emodin treatment was administered at concentrations of 5uM, 15uM, and 25uM for 2 h at 37 °C after the OGD/R model to determine the optimal therapeutic dose.

### Administration of Akt inhibitor Triciribin (TCN) and HO-1 inhibitor tin protoporphyrin IX (SNPP)

To further investigate the significance of the Akt or HO-1 pathway in II/R injury, II/R model animals were treated with the Akt inhibitor TCN (MedChemExpress, USA) at a dose of 0.5 mg/kg via the tail vein one hour before ischemia (*n* = 6). Similarly, Tin-protoporphyrin IX (SNPP), the HO-1 inhibitor obtained from MedChemExpress (USA), was dissolved in 0.1 mol/L NaOH to a concentration of 5 mM. It was then diluted in PBS and given to the mice (*n* = 6).

Meanwhile, to verify the potential mechanism in vitro experiment, the Akt inhibitor TCN was also administered at a dose of 10uM or the HO-1 inhibitor SNPP was administered at a dose of 20uM, 1 h prior to the OGD/R model.

### Histological morphological observation

Two independent and blinded researchers performed histological scoring of morphological damage using the Chiu histological injury scoring system for intestinal villi. The scoring system ranges from 0 (indicating normal mucosa) to 5 (representing loss of villi) as follows: Grade 0 = normal mucosa; Grade 1 = development of subepithelial Gruenhagen's spaces at the tip of the villi; Grade 2 = extension of subepithelial spaces with moderate lifting of the epithelial layer from the lamina propria; Grade 3 = massive epithelial lifting down the side of the villi; Grade 4 = completely denuded villi and dilated capillaries; Grade 5 = loss of villi, disintegration of the lamina propria, hemorrhage, and ulceration [27]. The assessment was carried out on 1 cm segments of intestinal specimens that had been embedded in paraffin and stained with hematoxylin and eosin. The 5 cm segment of the small intestine was removed from a location 5 cm proximal to the terminal ileum for subsequent biochemical tests.

### Wet/Dry weight ratio

Intestinal tissues were quantitatively removed and weighed to determine the wet weight. The dry weight was determined after drying the tissues in a 56 °C constant temperature box for 24 h. Finally, the ratio of the wet weight to dry weight was calculated to measure the water content of the intestinal tissue.

### Quantitative reverse transcription polymerase chain reaction (qRT-PCR)

The mRNA was extracted from intestinal tissues and Caco-2 cells to determine the transcription levels of inflammatory factors. The relative expression of pro-inflammatory cytokines, such as TNF, IL-6, IL-1β, and C-X-C Motif Chemokine Ligand 1 (CXCL1), were measured using the qRT-PCR detection system CFX96 (Bio-Rad, CA, USA), following the manufacturer's recommendations. Samples were homogenized in TRIzol reagent (Life Technologies, CA, USA), and total ribonucleic acid (RNA) was extracted using the RNeasy isolation kit (Qiagen, MD, USA) following the manufacturer's instructions. cDNA was synthesized from 200 ng of RNA using the Veriti™ 96-Well Fast Thermal Cycler (ThermoFisher Scientific, MA, USA). Next, the qRT-PCR reaction was performed on a CFX96 Real-Time PCR Detection System (Bio-Rad, CA, USA) using SYBR Green PCR Master Mix (Monad, Wuhan, China). The specific primers were designed using sequence data and nucleotide BLAST software from the National Center for Biotechnology Information database (http://www.ncbi.nlm.nih.gov/nucleotide) and were manufactured by Sangong Biotech (Shanghai, China) (Tables 1 and 2).

**Table 1:** Primers designed for qRT-PCR in animal experiment

Gene symbol	Forward primer	Reverse primer
β-actin	GTGACGTTGACATCCGTAAAGA	GCCGGACTCATCGTACTCC
IL-1β	CCGTGGACCTTCCAGGATGA	GGGAACGTCACACACCAGCA
TNF	CATCTTCTCAAAATTCGAGTGACAA	TGGGAGTAGACAAGGTACAACCC
IL-6	AGTTGCCTTCTTGGGACTGA	TCCACGATTTCCCAGAGAAC

**Table 2:** Primers designed for qRT-PCR in cell experiment

Gene symbol	Forward primer	Reverse primer
β-actin	CCTGGCACCCAGCACAAT	GGGCCGGACTCGTCATAC
IL-1β	ATGATGGCTTATTACAGTGGCAA	GTCGGAGATTCGTAGCTGGA
TNF	CCTCTCTCTAATCAGCCCTCTG	GAGGACCTGGGATAGATGAG
CXCL1	TGCAGGGAATTCACCCCAAGAAC	AGTGTGGCTATGACTTCGGTTTGG
IL-6	ACTCACCT CTTCAGAACGAATTG	CCATCTGAAGGTTCAGGTTG

### Measurement of MPO, SOD, and MDA

The intestinal tissues were collected 120 min after reperfusion to assess the levels of inflammation and oxidative stress. The activities of myeloid peroxidase (MPO), superoxide dismutase (SOD), and the level of malondialdehyde (MDA) in intestinal tissues were measured using commercial biochemical kits (Elabscience, Wuhan, China) following the manufacturer's instructions. The levels of MPO (E-BC-K074-S), SOD (E-BC-K019-S), and MDA (E-BC-K025-S) in the intestine were measured using spectrophotometry (Beckman, California, USA). Then, the optical density of MPO was recorded at 460 nm, MDA was determined at 532 nm, and SOD activity was measured at 550 nm. Finally, the MPO, MDA, and SOD contents were calculated using the formula provided in the manual.

### TUNEL staining

The apoptotic cells in the intestine and Caco-2 cells were detected using the terminal deoxynucleotidyl transferase-mediated dUTP nick end labeling (TUNEL) assay. We conducted TUNEL using an in situ cell death measurement kit (Servicebio, Wuhan, China) following the manufacturer's instructions. Staining with 4,6-diamino-2-phenyl indole (DAPI) (Servicebio, Wuhan, China) was performed to visualize the nuclei. Images were obtained using a fluorescent microscope (Olympus, Center Valley, USA). DAPI has an excitation wavelength of 330-380 nm and an emission wavelength of 420 nm, emitting blue light. FITC has an excitation wavelength of 465-495 nm and an emission wavelength of 515-555 nm, emitting green light.

### Western blot assay

Protein expressions in intestinal tissue (*n* = 3) and Caco-2 cells (*n* = 3) were monitored using western blot analysis. Briefly, the samples were processed using high-energy mixing with suitable grinding beads. Total protein extraction was performed using RIPA lysis buffer at 12,000 rpm in a microcentrifuge for 15 min at 4 °C. Protein concentration was determined, and sodium dodecyl sulfate–polyacrylamide gel electrophoresis (SDS-PAGE) was used to separate the proteins. The proteins were then transferred onto polyvinylidene difluoride (PVDF) membranes and blocked using 5% skim milk at 37 °C for 1 h. Next, the membranes were incubated overnight at 4 °C with specific primary antibodies, including Caspase-3 (Cell Signaling Technology, MA, USA), cleaved Caspase-3 (Cell Signaling Technology, MA, USA), Bax (Proteintech, Wuhan, China), Bcl-2 (Proteintech, Wuhan, China), p-Akt (Cell Signaling Technology, MA, USA), Akt (Cell Signaling Technology, MA, USA), HO-1 (Proteintech, Wuhan, China), and β-actin (Proteintech, Wuhan, China). Afterward, the membranes were washed three times with Tris-buffered saline with Tween (TBS-T) and incubated with a horseradish-conjugated goat anti-rabbit antibody (Cell Signaling Technology, MA, USA) at 37 °C for 1 h. Protein bands were visualized using the chemiluminescence (ECL) detection system (Bio-Rad, CA, USA), and protein quantification was performed using ImageJ (National Institutes of Health, USA). Protein expressions were normalized to β-actin as a reference.

### Statistical analysis

The measurement data were presented as mean ± standard deviation (SD). Statistical analysis was performed using SPSS 26.0 software (SPSS Inc., USA). One-way analysis of variance (ANOVA) was utilized and Tukey's multiple comparisons test was used for comparisons between groups. The figures were generated using GraphPad Prism software (version 9) by GraphPad Software, CA. Statistical significance was determined at a level of* P* < 0.05.

## Results

### Emodin improved intestinal barrier disruption, wet/dry weight ratio, inflammation, and oxidative stress in II/R injury mice

**Fig. 1:**
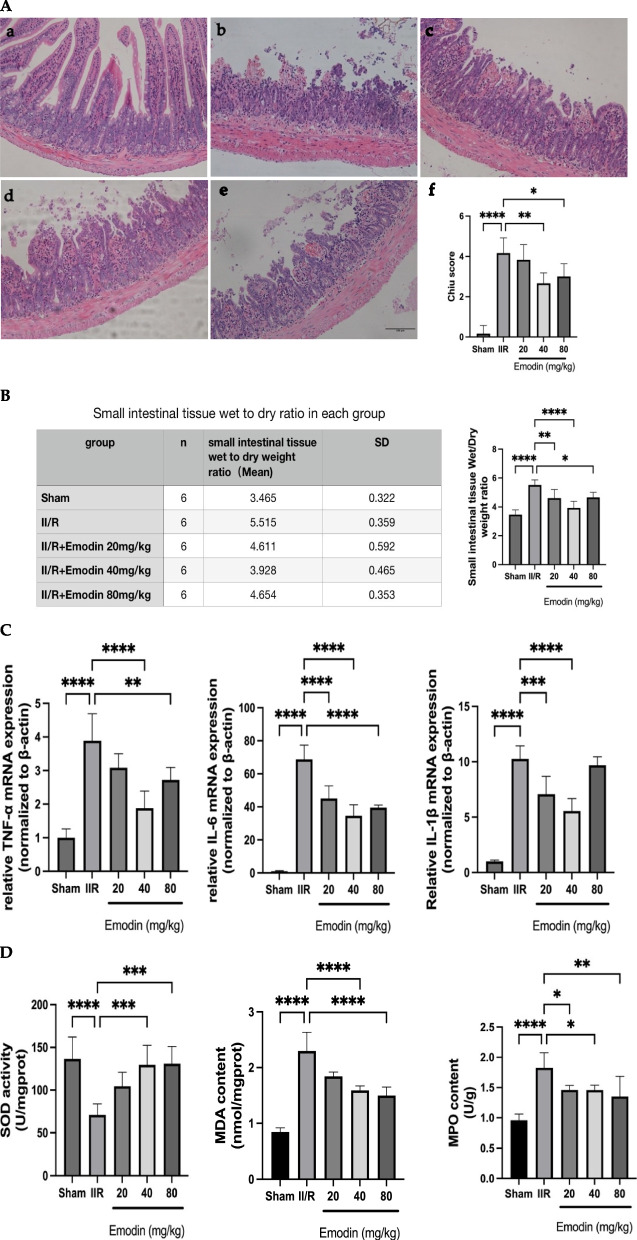
**A** Histological morphological changes of the intestine in H&E staining (scale bar = 100 μm) were shown in the following groups: **a** sham group, **b** II/R model group, **c** II/R plus 20 mg/kg Emodin, **d** II/R plus 40 mg/kg Emodin, **e** II/R plus 80 mg/kg Emodin, **f** Chiu score was used to measure the extent of intestinal damage. **B** Intestinal edema was measured using the wet/dry weight ratio of the intestine. The intestinal edema increased after II/R injury. However, after the use of Emodin with different doses, the intestinal edema decreased to varying degrees. **C** Different doses of Emodin alleviated intestinal inflammation caused by II/R injury. Pro-inflammatory factors such as IL-6, TNF and IL-1β were decreased after the use of Emodin. **D** Different doses of Emodin attenuated II/R-associated oxidative stress and inflammation to varying degrees. The 40 mg/kg Emodin and 80 mg/kg Emodin attenuated II/R-associated oxidative stress more effectively than 20 mg/kg Emodin

Histological examination revealed that injuries induced by II/R were characterized by villi shortening, loss of villous epithelium, and mucosal neutrophil infiltration. Administration of Emodin ameliorated these changes. The Chiu scoring and microphotographs were presented in Fig. 1A. No mucosal injury was observed in the sham group. II/R injuries were significantly more pronounced in the II/R model group compared to the sham group (*P* < 0.01). Administration of 40 mg/kg of Emodin significantly reduced the intestinal injury caused by II/R (*P* < 0.05). (Fig. 1A).

The wet/dry weight ratio increased in the II/R model group compared to the sham group (*P* < 0.01), indicating that the intestine underwent edema after II/R injury. Meanwhile, Emodin treatment effectively decreased the wet/dry weight ratio compared to the II/R model group to varying degrees. Among the different treatment doses, the II/R plus 40 mg/kg Emodin group showed the greatest alleviation of intestinal edema (*P* < 0.0001). (Fig. 1B).

The concentrations of TNF, IL-1β, and IL-6 were significantly elevated in the intestinal tissue of II/R model mice compared to the sham group (all *P* < 0.0001). Furthermore, administration of Emodin protected against II/R injury and reduced the levels of TNF, IL-1β, and IL-6 in the intestinal tissue compared to the II/R injury group (all *P* < 0.01). (Fig. 1C).

Figure 1D illustrated that the level of MDA was higher in the intestinal tissue of II/R model mice compared to the sham group (*P* < 0.0001). More importantly, treatment with Emodin significantly decreased the levels of MDA in the intestinal tissue of mice subjected to II/R injury with statistical significance. Furthermore, the levels of superoxide dismutases (SOD), an antioxidant agent, decreased significantly in the II/R model group compared to the sham group (P < 0.0001). Meanwhile, administration of Emodin elevated the SOD activity compared to II/R model group. MPO level, which is a crucial inflammatory enzyme, showed a significant increase in the intestinal tissues of the II/R model group compared to the sham group (P < 0.0001). Administration of Emodin resulted in a significant decrease in MPO level in II/R mice (P < 0.05).

### Emodin mitigated cell apoptosis and regulated the levels of cleaved Caspase-3/Caspase-3, Bcl-2, Bax, p-Akt/Akt, and HO-1 in II/R mice

**Fig. 2:**
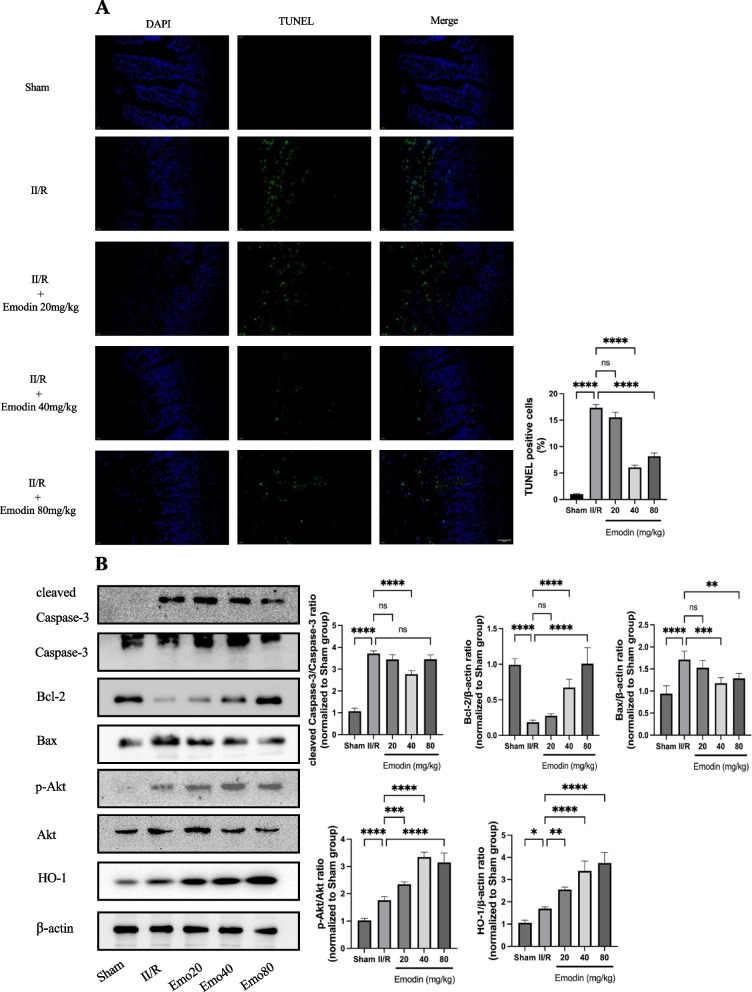
**A** Different doses of Emodin attenuated II/R-associated apoptosis. The Emodin doses of 40 mg/kg and 80 mg/kg significantly attenuated II/R-associated apoptosis compared to the 20 mg/kg Emodin dose. **B** Different doses of Emodin inhibited apoptosis by down-regulating pro-apoptosis associated proteins such as cleaved Caspase-3/Caspase-3 and Bax, and up-regulating anti-apoptosis associated protein such as Bcl-2. Emo20: II/R plus 20 mg/kg Emodin, Emo40: II/R plus 40 mg/kg Emodin, Emo80: II/R plus 80 mg/kg Emodin. Quantitative analysis of the expression compared with β-actin was shown beside

In order to explore whether Emodin can alleviate intestinal injury in II/R mice by inhibiting apoptosis, we assessed the apoptosis of intestinal tissues. Notably, apoptotic chromatin condensation was significantly observed in the II/R model group compared to the sham group (*P* < 0.0001). This indicated that intestinal epithelial cells underwent significant apoptosis after exposure to II/R. Pretreatment with Emodin significantly inhibited the apoptosis of intestinal tissue induced by II/R. (Fig. 2A).

Meanwhile, the apoptosis-executing proteins, cleaved Caspase-3/Caspase-3 and Bax, were increased in II/R mice. And there was an obvious decrease in these proteins after pretreatment with Emodin. The anti-apoptotic protein, Bcl-2, was decreased in II/R mice, and it showed a significant increase after pretreatment with Emodin. To add to this, p-Akt/Akt and HO-1 were also increased in mice with II/R injury and showed a significant increase after pretreatment with Emodin (*P* < 0.05). The findings showed that the role of Emodin in improving II/R injury was possibly related with Akt/HO-1 signaling pathway. (Fig. 2B).

### The protective effects of Emodin against II/R injury were reversed when Akt or HO-1 was inhibited in mice

**Fig. 3:**
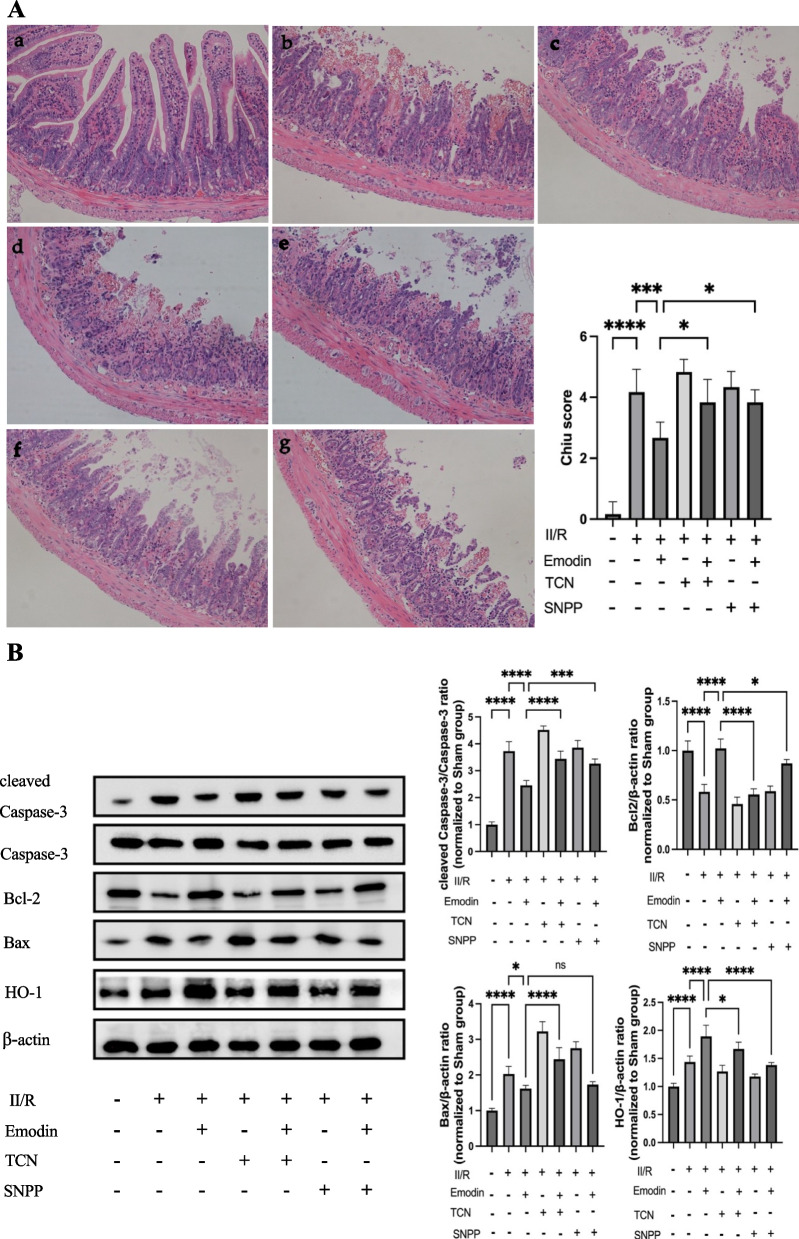
**A** H&E staining in intestinal tissue: **a** sham group, **b** II/R model group, **c** 40 mg/kg Emodin treatment group, **d** TCN treatment before II/R, **e** Treatment with 40 mg/kg of Emodin following TCN treatment before II/R, **f** SNPP treatment before II/R, **g** Treatment with 40 mg/kg of Emodin following SNPP treatment before II/R. **B** Akt and HO-1 inhibitor administration effectively counteracted the protective effects of Emodin in mitigating II/R injury and promoted the occurrence of apoptosis. Emodin represents II/R plus 40 mg/kg Emodin. Quantitative analysis of the expression compared with β-actin, as shown alongside

As shown in Fig. 3A, the intestinal Chiu score was significantly higher in the II/R model group compared to the sham group (*P* < 0.0001). However, pretreatment with Emodin resulted in a decrease in the intestinal Chiu score (*P* < 0.001). We further used Akt or HO-1 inhibitors before administering Emodin to the treatment group. The changes in intestinal pathology showed that the administration of Akt or HO-1 inhibitors significantly reversed the protection provided by Emodin against II/R injury to some extent, respectively (*P* < 0.05).

Figure 3B showed that protein levels of HO-1 in the intestine were significantly increased after II/R injury (*P* < 0.05). Treatment with Emodin further resulted in a significant upregulation of HO-1 protein expression (*P* < 0.05), consistent with the findings observed in Fig. 2B. Meanwhile, Akt inhibition significantly decreased HO-1 expression (*P* < 0.05), indicating that Emodin may protect against II/R injury through Akt/HO-1 signaling pathway.

These findings indicated that Emodin inhibited intestinal injury mediated by II/R, possibly by regulating Akt or HO-1, at least to some extent. Later, Akt or HO-1 inhibition was administered before the II/R injury model group received Emodin treatment individually. The results showed that Akt or HO-1 inhibition before Emodin treatment significantly increased the levels of pro-apoptosis proteins, such as cleaved Caspase-3/Caspase-3 and Bax, and decreased the levels of anti-apoptosis protein, such as Bcl-2, compared to the Emodin treatment group. Pointing out that inhibiting Akt or HO-1 significantly reversed the protective effects of Emodin against apoptosis in mice with II/R injury. Interestingly, II/R mice with Akt or HO-1 inhibition showed decreased HO-1 expression simultaneously, suggesting that Akt regulates HO-1. Additionally, Emodin treatment resulted in a partial increase in HO-1 expression.

### Emodin alleviated cell inflammation, inhibited cell apoptosis, and regulated the levels of cleaved Caspase-3/Caspase-3, Bcl-2, Bax, p-Akt/Akt, and HO-1 in OGD/R-induced Caco-2 cells

**Fig. 4:**
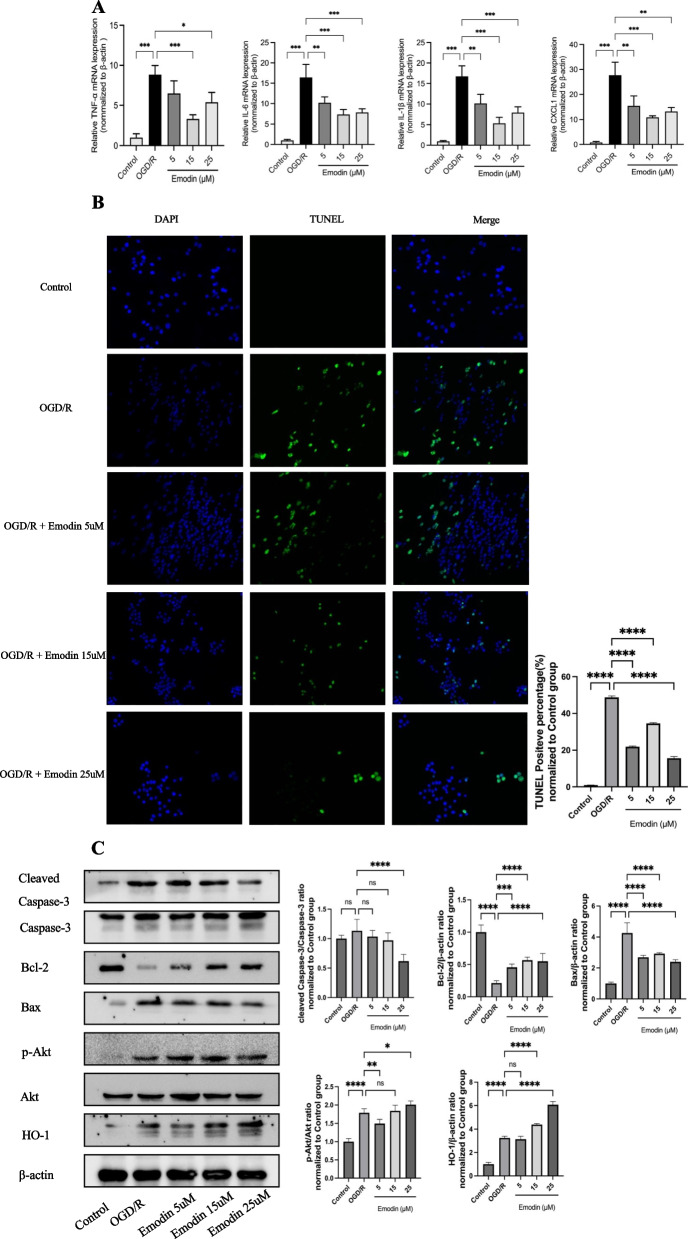
**A** Pro-inflammatory cytokines, including TNF, IL-6, IL-1β, and CXCL1, were decreased following the administration of Emodin. **B** Different doses of Emodin attenuated OGD/R-associated apoptosis. **C** Different doses of Emodin inhibited OGD/R-induced apoptosis by downregulating pro-apoptosis-associated proteins such as cleaved Caspase-3/Caspase-3 and Bax, while upregulating anti-apoptosis-associated proteins like Bcl-2. Proteins that regulate apoptosis, such as p-Akt/Akt and HO-1, were also altered accordingly. Quantitative analysis of the expression compared with β-actin was shown beside

The inflammation levels of TNF, IL-1β, IL-6, and CXCL1 in Caco-2 cells showed a significant increase following OGD/R (*P* < 0.001). Treatment with 15 and 25 μM Emodin effectively reduced the damage caused by OGD/R (all *P* < 0.05). The treatment with 15 μM Emodin demonstrated the most significant efficacy (*P* < 0.001). Therefore, 15 μM of Emodin was used for all subsequent experiments. (Fig. 4A).

The TUNEL staining of Caco-2 cells showed a significant increase in the percentage of TUNEL-positive cells following OGD/R (*P* < 0.0001). Treatment with Emodin effectively inhibited the damage caused by OGD/R (all *P* < 0.0001). (Fig. 4B).

The apoptosis executing proteins, cleaved Caspase-3/Caspase-3 and Bax, were increased in OGD/R induced Caco-2 cells, which experienced an obvious decrease after pretreating with Emodin. The anti-apoptotic protein, Bcl-2, was decreased in Caco-2 cells induced by OGD/R, but it showed a significant increase after pretreatment with Emodin. In addition, the levels of apoptosis regulatory factors, p-Akt/Akt, and HO-1, were also found to be increased in Caco-2 cells induced by OGD/R. After treatment with Emodin, these factors showed a significant elevation (*P* < 0.05). The findings showed that the role of Emodin in improving OGD/R injury was closely related to its anti-apoptotic effect and the possibly regulating pathway was Akt/HO-1 signaling. (Fig. 4C).

### The protective effect of Emodin against apoptosis following OGD/R was eliminated when Akt or HO-1 was inhibited in Caco-2 cells

**Fig. 5:**
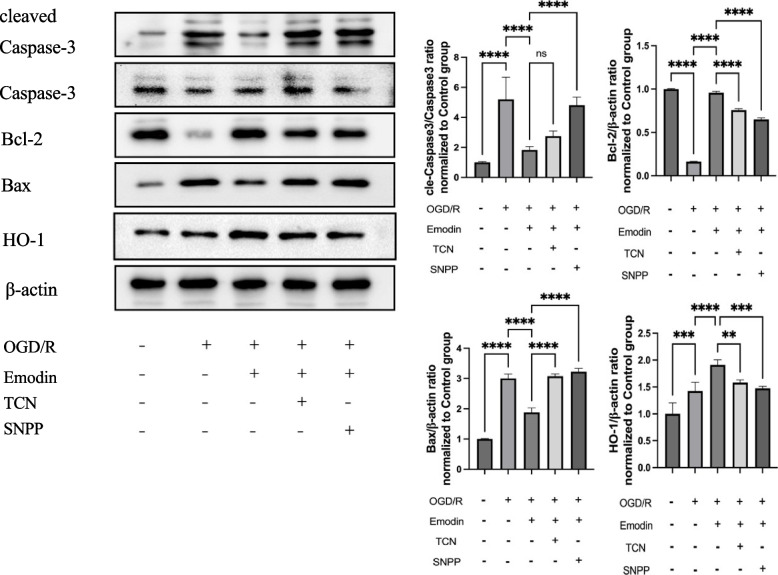
The protective effect of Emodin against apoptosis following OGD/R was eliminated when Akt or HO-1 was inhibited in Caco-2 cells. This included the levels of cleaved Caspase-3/Caspase-3, Bcl-2, Bax, and HO-1 in OGD/R Caco-2 cells. Emodin represents OGD/R plus 15 μM Emodin. Quantitative analysis of the expression compared with β-actin was shown next to it

To further investigate the protective mechanism of Emodin against OGD/R in Caco-2 cells. We also separately inhibited Akt or HO-1 in Caco-2 cells induced by OGD/R using TCN and SNPP. Results showed that Emodin treatment significantly reduced apoptosis in Caco-2 cells induced by OGD/R (all *P* < 0.01). Meanwhile, the protective effects of Emodin were reversed with statistical significance when Akt and HO-1 were inhibited. Interestingly, in groups injected with the Akt inhibitor, the level of HO-1 decreased, indicating that Akt can regulate the level of HO-1. (Fig. 5).

## Discussion

Acute II/R injury is a common clinical disorder characterized by a significant reduction in intestinal blood flow, leading to bowel necrosis and high mortality rates [28]. It can occur in different clinical scenarios, including abdominal surgeries, hemorrhagic shock, and cardiopulmonary bypass surgery [29, 30]. Apart from causing direct damage to the intestinal tissue, II/R injury can also induce an inflammatory response and oxidative stress in distant organs or throughout the body. This can ultimately lead to lung damage [31] and multiple organ failure.

Here, we described the protective effect of Emodin on II/R injury. Emodin was found to alleviate the disruption of the intestinal epithelial barrier, decrease oxidative stress, and inhibit inflammation. Furthermore, Emodin helped maintain the integrity of the intestinal epithelial barrier by promoting anti-apoptotic proteins like Bcl-2 and inhibiting pro-apoptotic proteins such as cleaved-Caspase-3/caspased3 and Bax. Besides, our study also confirmed that Emodin could alleviate II/R injury through the Akt/HO-1 signaling pathway.

To our knowledge, oxidative stress and apoptosis are primary driving force in the development of II/R injury [5, 8]. Increasing numbers of studies have shown that Emodin, a naturally occurring anthraquinone, has well-established biological properties [32, 33]. Besides its anti-infection effects, Emodin treatment also exhibited organ protection properties, including neuroprotection, hepatoprotection, and antiallergic activities [34]. A recent study by Lu et al. found that Emodin prevented renal I/R injury by suppressing p53-mediated cell apoptosis [35]. Our study partially provided some insight into the effects of Emodin on regulating intestinal dysfunction during I/R injury.

In the mouse model of II/R injury, we assessed the protective role of Emodin in maintaining the intestinal barrier functions through reducing oxidative stress, suppressing inflammation, and preventing apoptosis. These findings were in consistent with previous studies conducted by Gao et al. and Song et al., which demonstrated that Emodin effectively reduced oxidative stress, inhibited inflammation, and improved intestinal barrier functions [36–38]. In other animal models, Emodin was found to alleviate intestinal barrier dysfunction by inhibiting apoptosis and help restore intestinal epithelial tight junction barrier integrity [39, 40]. We also confirmed that Emodin inhibited inflammation and apoptosis in OGD/R-induced Caco-2 cell model. In accord with our results, Tu’s group showed that Emodin inhibited lipopolysaccharide-induced inflammation in cells [41] and Zheng et al. discovered that Emodin protected against cell apoptosis through the PI3K/AKT/mTOR pathway in human hepatocytes [42].

It is worth noting that prior research has indicated that Emodin can potentially have both enhancing and inhibitory impacts on the apoptotic response triggered by II/R injury. In accordance with our experiment, the beneficial effect against apoptosis in intestinal injury was verified in a severe acute pancreatitis model [39]. Additionally, Emodin treatment distinctly attenuated the apoptosis of intestinal tissues induced by acute intestinal injury [22]. In contrast, some studies showed that Emodin induced apoptosis and suppressed the growth of non-small-cell lung cancer cells, demonstrating an anti-tumor mechanism by inhibiting proliferation [43].

Furthermore, AMPK/Akt is a crucial transcription factor that regulates inflammation and oxidative stress responses in cells [44]. It modulates the expression of genes that encode HO-1 and other antioxidative enzymes, playing a crucial role in the preventive mechanism against oxidative stress [45]. Through inhibiting HO-1, we observed a significant increase in pro-apoptotic proteins and a decrease in anti-apoptotic proteins. Moreover, we observed that the inhibition of Akt resulted in a decrease in HO-1 expression and a corresponding change in apoptotic proteins, indicating a significant regulatory role of Akt in modulating HO-1.

In conformity with our research, recent evidence indicates that Emodin has the potential to reduce damage by regulating the Akt signaling pathway. According to the research, studies suggested that Emodin could enhance the recovery from LPS-induced myocardial injury by activating the PI3K/Akt pathway. Additionally, Emodin could protect against homocysteine-induced cardiac dysfunction by reducing oxidative stress through the Akt/eNOS/NO signaling pathways [46, 47]. Moreover, the protective role of Akt signaling against I/R injury has also been extensively explored [48]. Similarly, the underlying mechanism of Emodin's impact on HO-1 signaling has also been investigated. Gao et al. confirmed that Emodin protected against acute pancreatitis-associated lung injury by inhibiting NLPR3 inflammasome activation via the Nrf2/HO-1 signaling pathway; and Shang et al. also illustrated Emodin's protective effects against sepsis-associated damage to the intestinal mucosal barrier through the VDR/Nrf2/HO-1 pathway [49, 50]. These studies highlighted the underlying mechanism of Emodin's impact on HO-1 signaling. To add to this, the HO-1 signaling pathway also effectively provides protection against I/R injury [51]. Furthermore, accumulating evidence from preclinical studies suggested that the Akt/Nrf2/HO-1 signaling pathway protects against oxidative stress, inflammation, and apoptosis [52–54]. The AMPK/Akt signaling pathway, which was activated by oxidative stress and inflammation, led to the activation of NRF2/HO-1 [55]. And this beneficial effect was nullified by silencing NRF2 [56].

In contrast to our study, there is evidence to suggest that the activation of Akt signaling can actually worsen I/R injury. Some research showed that inhibition of PTEN/Akt/mTOR signaling pathway decreased cardiac I/R injury free from excessive autophagy [57]. As HO-1 can have both protective and deleterious effects in I/R injury, and its roles are determined by the levels of enzyme expression. In some studies, it was observed that, in contrast to our own research results, the levels of HO-1 were actually found to rise during the period of I/R when hyperthermia was present. Additionally, there was a faster decline in the number of astrocytes and neurons in the hippocampus of gerbils [58].

Although this study has unveiled a number of significant findings, there are also limitations. This study has only examined the effect of Emodin and its underlying mechanism in animal experiments and cell models. It should be pointed out that we did not collect clinical data in this research. Our research group will include clinical data in future studies to further investigate its clinical significance and potential targets.

## Conclusions

This study demonstrates the protective role of Emodin in mitigating II/R injury in the small intestine. Emodin achieves this protection by reducing inflammation and oxidative stress, potentially through the Akt-mediated HO-1 upregulation.

## Data Availability

No datasets were generated or analysed during the current study.
